# Exploring an Odor-Baited “Trap Bush” Approach to Aggregate Plum Curculio (Coleoptera: Curculionidae) Injury in Blueberries

**DOI:** 10.3390/insects10040113

**Published:** 2019-04-19

**Authors:** Cesar Rodriguez-Saona, Anne Nielsen, David Shapiro-Ilan, Sunil Tewari, Vera Kyryczenko-Roth, Nicolas Firbas, Tracy Leskey

**Affiliations:** 1 P.E. Marucci Center for Blueberry & Cranberry Research, Rutgers University, Chatsworth, NJ 08019, USA; vera.kyryczenko@rutgers.edu (V.K.-R.); nicolas.firbas@gmail.com (N.F.); 2 Rutgers Agricultural Research and Extension Center, Rutgers University, Bridgeton, NJ 08302, USA; nielsen@njaes.rutgers.edu; 3 USDA-ARS, Fruit and Tree Nut Research Laboratory, Byron, GA 31008, USA; david.shapiro@ars.usda.gov; 4 Corteva Agriscience, Fresno, CA 93706, USA; sunil.tewari@corteva.com; 5 USDA-ARS, Appalachian Fruit Research Station, Kearneysville, WV 25430, USA; tracy.leskey@ars.usda.gov

**Keywords:** *Conotrachelus nenuphar*, *Vaccinium corymbosum*, weevil, semiochemicals, aggregation pheromone

## Abstract

This 2-year study (2013–2014) assessed the efficacy of an odor-baited “trap bush” approach to aggregate plum curculio, *Conotrachelus nenuphar*, adult injury, i.e., number of oviposition-scared fruit, in four commercial highbush blueberry farms in New Jersey (USA). In each farm, we compared fruit injury in bushes baited with grandisoic acid and benzaldehyde along the perimeter of trap-bush plots versus unbaited bushes in control plots. We also measured the amount of fruit injury in neighboring bushes (i.e., spillover effect) and in the plots’ interior. In both years, the amount of fruit injury by *C. nenuphar* adults was greater on and near odor-baited bushes in trap-bush plots compared with those on and near unbaited bushes in control plots, indicative of aggregation. Injury in unbaited bushes neighboring trap bushes was often greater than unbaited bushes in control plots, providing some evidence for a spillover effect. However, no difference in fruit injury was found between interior trap-bush and control plots. Therefore, odor-baited trap bushes can be used in blueberries to manipulate *C. nenuphar* foraging behavior, i.e., aggregate adults, without compromising injury in field interiors. Under this approach, insecticides could then be targeted at only a few (perimeter-row) bushes within fields rather than entire fields.

## 1. Introduction

Native to North America, the plum curculio, *Conotrachelus nenuphar* (Herbst) (Coleoptera: Curculionidae), is a key pest of stone and pome fruits (e.g., apples, peaches, nectarines, and cherries) and blueberries in the eastern United States [1,2,3,4]. Damage from this pest can reach nearly 100% annually in apples if left unchecked [5]. Conventional growers have relied upon broad-spectrum insecticides like organophosphates to provide commercially acceptable levels of control [6,7,8]. In New Jersey (USA), *C. nenuphar* is considered one of the most important fruit-feeding pests of blueberries [9]. Adults move into blueberry fields in early spring (mid-April through May) from nearby overwintering sites and their activity peaks during bloom [9], when bees are active. This limits the use of conventional control tactics until bee removal and makes early and mid-season varieties more susceptible to oviposition injury [10]. Therefore, if needed based on numbers of adults on bushes and injured fruit, a single post-bloom insecticide application is typically directed at the adults of this pest [10,11]. A single egg is laid on young, green blueberry fruit, larvae develop inside the fruit, and then exit the fruit to pupate in the soil [9]. The next-generation adults emerge in July–August and soon after move out of the fields to overwinter in the surrounding wooded habitats [9].

In blueberries, the use of broad-spectrum insecticides, i.e., organophosphates, is being reduced as a result of the food quality protection act (FQPA) and due to maximum residue limits (MRLs) set for exporting fruit [12]. Growers are, to varying degrees, reducing the amount of these insecticides applied and/or replacing them with newer reduced-risk materials that, while effective, are more costly [13]. For example, two reduced-risk insecticides, novaluron (Rimon^®^; Makhteshim Agan of North America, Inc., Raleigh, NC, USA) and indoxacarb (Avaunt^®^; DuPont, Wilmington, DE, USA), have recently been registered for pre-bloom and post-bloom control of *C. nenuphar*, respectively, in blueberries [11]. More precise management strategies based on behavior manipulation of *C. nenuphar* can be critical for successful implementation of a narrow-spectrum, reduced-spray program as opposed to conventional, whole-field insecticide applications.

Currently, in blueberries, *C. nenuphar* adults are monitored using beat-sheet sampling [9]. However, adults are cryptically colored and active mainly at night [4,14,15,16], which makes it difficult to monitor them using this method. Recently, Hernandez-Cumplido et al. [17] showed that pyramid traps baited with a synergistic two-component lure, comprised of the synthetic host plant-derived volatile benzaldehyde [18] and the synthetic male-produced aggregation pheromone grandisoic acid [19], are effective for monitoring *C. nenuphar* adults in blueberries. In apple orchards, an effective monitoring approach has been the “trap-tree” method [20,21,22], which is based on baiting selected perimeter row apple tree canopies with the synergistic lure composed of benzaldehyde and grandisoic acid [23]. This method results in aggregation of *C. nenuphar* adults within the canopies of odor-baited trees. These trap trees are subsequently monitored for signs of fresh oviposition injury, thereby allowing growers to determine the need for, and timing of, insecticide applications [20,21]. The effectiveness of this approach for monitoring oviposition activity within trap trees as a biological trigger for insecticide applications has been validated in seven northeastern states [22]. Based on these studies, the odor-baited trap-tree approach was also applied as a management tool for *C. nenuphar* in apples [24]. In this case, the trap-tree approach is used to aggregate *C. nenuphar* adults in specific baited perimeter-row trees. Then, insecticides are applied only to those baited trap-trees located on the periphery of apple orchards, rather than the entire perimeter row or the entire orchard (after petal fall) [24]. Prior to our research, however, the trap-tree approach had never been tested in fruit crops other than apples.

Studies were conducted in 2013 and 2014 in commercial highbush blueberry, *Vaccinium corymbosum* L., farms in New Jersey to test the trap-tree (referred to here as “trap bush”) approach for aggregating *C. nenuphar* oviposition activity. We hypothesized that this approach will result in aggregated oviposition injury in and around odor-baited perimeter blueberry bushes, resulting in reduced fruit injury in field interiors. Specifically, we addressed whether the presence of synthetic baits in blueberry bushes results in: (1) significant aggregation of fruit injury in these specific bushes compared with unbaited bushes; (2) greater fruit injury in neighboring bushes compared with unbaited bushes as a result of a spillover effect [24]; and (3) lower *C. nenuphar* fruit injury in baited field interiors compared with unbaited fields.

## 2. Materials and Methods

### 2.1. Sites and Study Design

**Figure 1:**
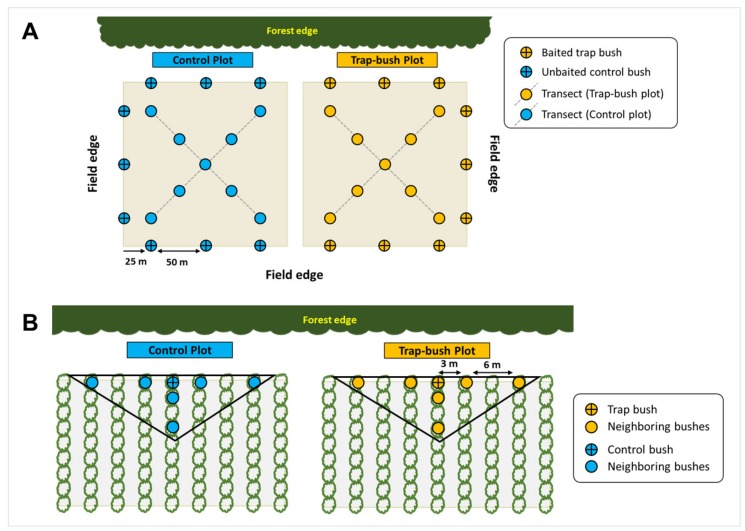
Layout of paired odor-baited trap-bush and unbaited (control) plots in commercial highbush blueberry farms (**A**). Sampling regimes used to compare fruit injury in trap bush and control plots. Each plot was ~2.5 ha.

The study was conducted in 2013 and 2014 in eight experimental plots across four commercial blueberry farms (two paired plots per farm) located in Hammonton, New Jersey (Farm A: 39°33′23.48″N, 74°46′35.64″W; Farm B: 39°32′45.06″N, 74°47′19.47″W; Farm C: 39°35′37.50″N, 74°46′21.21″W; Farm D: 39°38′35.37″N, 74°40′22.14″W). These plots had the early blueberry variety “Duke” and were chosen based on the previous history of *C. nenuphar* infestation. Each plot was ~2.5 ha, with one of the perimeter rows bordering a wooded edge. Both plots within each farm were managed in the same manner, and decisions regarding insecticides followed recommendations by the Commercial Blueberry Pest Control Recommendations for New Jersey [11]. Within each farm, one of the plots was randomly assigned as the “trap-bush” plot; while the other plot was the “control.” In the trap-bush plots, nine perimeter row bushes (three bushes in each of the three perimeter rows of the plot not shared with the control plot; Figure 1A) were baited with one dispenser of benzaldehyde and one dispenser of grandisoic acid. A single benzaldehyde was used because of the smaller canopy size of blueberries compared to apples (in apple, four benzaldehyde dispensers are used per trap tree; e.g., [22,24]). The benzaldehyde dispenser consisted of 15-mL capped polyethylene vials (PGC Scientific Corp., Gaithersburg, MD, USA) containing 8 mL of a 9:1 solution of benzaldehyde: 1,2,4-trichlorobenzene (Sigma-Aldrich, St. Louis, MO, USA), as a stabilizing agent [25], for a release rate of ~10 mg/d [23]. Each vial was suspended inside an inverted colored (red) plastic drinking Solo^®^ cup (266 mL; Solo Cup Co., Urbana, IL, USA) to protect the stability of benzaldehyde from the potential negative impact of UV light. The pheromone dispenser contained 35 mg of grandisoic acid (ChemTica, San Jose, Costa Rica), with a release rate of ~0.14 mg/d [26]. The benzaldehyde dispensers were deployed at the center of each bush and left for the entire season, while the pheromone dispenser was also deployed near the center of the bush but replaced once after ~3 weeks. Within trap-bush plots, the first trap bush was deployed ~25 m from the end of the row, and trap bushes were separated by ~50 m within the row (Figure 1A). Bushes in control plots did not receive either benzaldehyde or pheromone dispensers. All trap-bush treatment plots were established during the 1st week of May, which coincided with mid-bloom and *C. nenuphar* adult peak activity in blueberries [17]. The same farms and plots were used in both years.

### 2.2. Fruit Evaluation

We collected fruit samples to address: (1) whether trap bushes result in significant aggregation of fruit injury in the specific trap bushes compared with control bushes, (2) whether fruit injury is greater in bushes neighboring trap bushes compared with control bushes (i.e., spillover effect), and (3) whether injury in plot interiors differs between trap-bush and control plots. For #s 1 and 2, the total number of fruit with oviposition scars was recorded from a sample of six random clusters per bush ( ~40 berries per bush) from odor-baited trap bushes in the trap-bush plots and from unbaited bushes in the control plots, and from six peripherally located trees surrounding each trap bush and control bush (Figure 1B), for a total of approximately 2500 fruit per plot. Three of the sampled peripheral bushes were next to the trap bush (“near” neighbors; i.e., 1.5–3 m away from the trap bush), while the other three were two bush-lenghts away (“far” neighbors; i.e., 4-6 m away from the trap bush) (Figure 1B). In both years, fruit samples were taken in two dates: the 1st (pre-spray) samples were taken at early fruit set and before any insecticide application; the 2nd (post-spray) samples were taken 10–14 days later and after a post-bloom insecticide application of phosmet (Imidan^®^; Gowan Co., Yuma, AZ, USA). In 2013, fruit injury evaluations were made on 21–23 May (pre-spray samples) and on 4–6 June (post-spray samples); while in 2014, fruit injury evaluations were made on 23–27 May (pre-spray samples) and on 3–6 June (post-spray samples). Prior to bloom, all plots were treated with novaluron (Rimon^®^) on 17–18 April. All plots were also treated with phosmet post-bloom on 28–30 May, after honeybee hives had been removed from the fields. Phosmet has both curative, i.e., reduces larval emergence rates, and adulticidal activity, while novaluron has anti-ovipositional/ovicidal activity [27,28]. Timing of the post-bloom insecticide application was based on injury data obtained from fruit samples across both plots within farms.

Additional fruit samples were taken to determine if aggregation of injury in trap bushes results in lower fruit injury in the interiors of trap-bush plots compared with the interiors of control plots. For this, approx. 360 berries were sampled from nine interior bushes (Figure 1A) in the trap-bush and control plots. Therefore, a combined total of ~5760 berries was taken from trap-bush and control plots (40 berries × 18 bushes × 4 plots) for each of the two sampling dates and the two years.

### 2.3. Data Analyses

Prior to analyses, the proportion of scarred fruit per bush was calculated. Data were analyzed separately for each of the two years (2013 and 2014) and for each of the two fruit sampling dates (pre- and post-sprays). Data were analyzed separately for each sampling date within year because, although not significant, the mean proportion of oviposition-scarred fruit was 32% higher before than after the post-bloom insecticide sprays (pre-spray: 2.49% ± 0.38% injured fruit; post-spray: 1.88% ± 0.28% injured fruit; Welch *t*-test_244.94_ = −1.33, *p* = 0.186). We also combined data from all neighboring bushes prior to analysis because only in one out of the four sampling dates was the amount of injured fruit significantly higher in neighboring bushes “near” the trap bushes than in neighboring bushes “far” from the trap bushes (mean ± SE percent of injured fruit in “near” and “far” neighbors for: 2013 pre-spray sample = 3.21 ± 0.34 and 2.41 ± 0.32, respectively (Welch *t*-test_411.86_ = 1.71, *p* = 0.044); 2013 post-spray sample = 1.28 ± 0.17 and 1.1 ± 0.15 (Welch *t*-test_412.95_ = 0.78, *p* = 0.218); 2014 pre-spray sample = 0.94 ± 0.14 and 0.79 ± 0.14 (Welch *t*-test_419.61_ = 0.78, *p* = 0.217); 2014 post-spray sample = 0.96 ± 0.16 and 0.98 ± 0.15 (Welch *t*-test_419.12_ = −0.08, *p* = 0.534).

First, we used Generalized Linear Models (GLMs) to determine the effects of baiting a bush with our synergistic lure and its proximity to the forest on the proportion of scarred fruit. Our full models included the independent variables: “Treatment” (trap bush versus control), “Location” (forest edge versus interior edge), “Farm”, and the 2- and 3-way interactions among them. “Farm” was included as a random effect. Second, to specifically address our hypotheses that the trap bush approach results in aggregated oviposition injury in (Hypothesis 1) and around (Hypothesis 2) baited blueberry bushes, which results in reduced fruit injury in field interiors (Hypothesis 3), we used Welch’s two-sample *t*-tests to compare the following: (1) whether the mean proportion of scarred fruit in trap bushes differs from the mean proportion of scarred fruit in control bushes (Hypothesis 1); (2) whether the mean proportion of scarred fruit in bushes neighboring trap bushes differs from the mean proportion of scarred fruit in bushes neighboring control bushes (as further evidence for a spillover effect) (Hypothesis 2a); (3) whether the mean proportion of scarred fruit in trap bushes differs from the mean proportion of scarred fruit in bushes neighboring trap bushes in trap bush plots (as evidence for an spillover effect) (Hypothesis 2b). For comparison, we also determined if the mean proportion of scarred fruit in unbaited bushes differs from the mean proportion of scarred fruit in bushes neighboring unbaited bushes in control plots; (4) whether the mean proportion of scarred fruit in interior bushes from trap-tree plots differs from the mean proportion of scarred fruit in interior bushes from control plots (Hypothesis 3).

These comparisons were done separately for each sampling date in both years. We also used Welch tests to test for differences in the levels of fruit injury between “near” and “far” bushes neighboring the trap bushes. The Welch tests account for unequal variances. As data on the proportion of scarred fruit per fruit were close to zero values, there was no need to use the arcsine square root transformation [29]. For clarity, data are shown as percentages (instead of proportions) in Results and Figures. All analyses were performed in R software version 3.4.3 [30].

## 3. Results

Across all plots, the mean proportion of *C. nenuphar* oviposition-scarred fruit was three times higher in 2013 than in 2014 (2013: 3.26% ± 0.38% (mean ± SE) injured fruit; 2014: 1.12% ± 0.23% injured fruit; Welch *t*-test_216.18_ = 4.73, *p* < 0.001).

### 3.1. Does Fruit Injury Differ Between Trap and Control Bushes (Hypothesis 1)?

**Figure 2:**
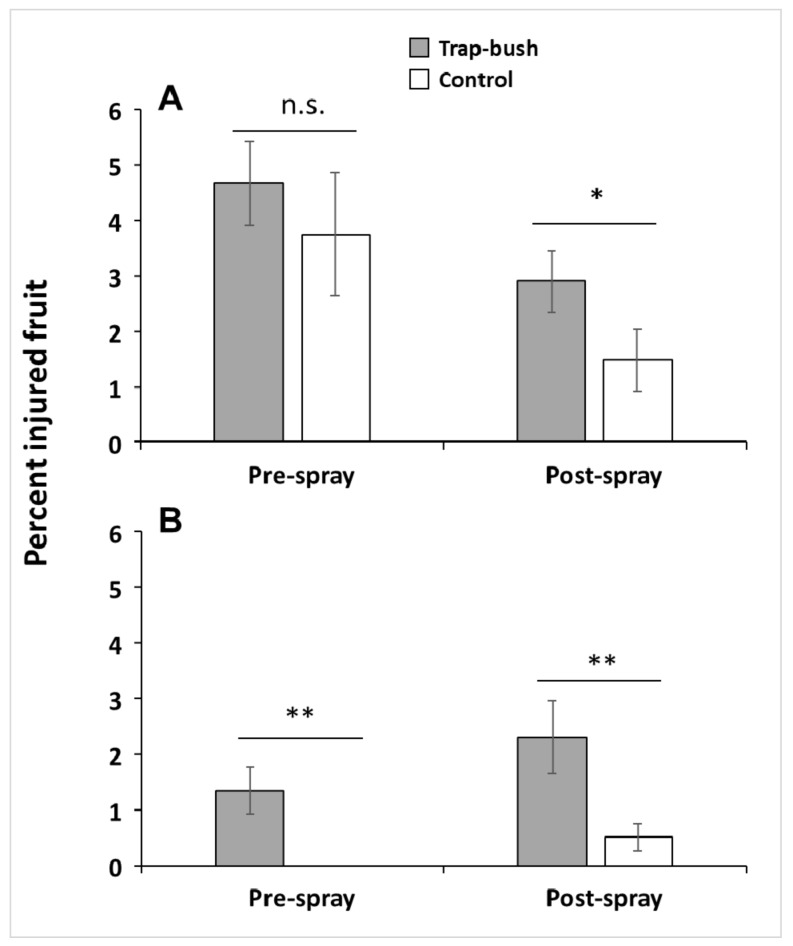
Percent (mean ± SE) of oviposition-scarred fruit by *Conotrachelus nenuphar* adults in odor-baited trap bushes and unbaited bushes in trap-bush and control plots, respectively, in 2013 (**A**) and 2014 (**B**). Fruit injury was assessed before and after a post-bloom insecticide application. Asterisks indicate significant differences according to Welch’s two-sample *t*-test (* = 0.01< *p* ≤ 0.05; ** = *p* ≤ 0.01); n.s. = not significant (*p* > 0.05).

Baiting blueberry bushes with the combination of grandisoic acid and benzaldehyde had a significant effect on the mean proportion of *C. nenuphar* oviposition-scarred fruit in 2013 (post-spray sample) and 2014 (pre- and post-spray samples) (significant “Treatment” effect; Table 1). The level of injured fruit was 2 and 4.5 times higher in trap bushes than in unbaited bushes in the 2013 (Welch *t*-test_63.67_ = −1.82, *p* = 0.037) and 2014 (Welch *t*-test_45.75_ = −2.59, *p* = 0.006) post-spray samples, respectively (Figure 2). In the 2014 pre-spray sample, injured fruit was only detected in trap bushes and not in unbaited bushes (Welch *t*-test_36_ = −3.21, *p* = 0.001) (Figure 2).

Bushes along plot edges facing the forest also had more fruit injury than interior bushes in the 2013 pre-spray sample, but not in any of the other sampling dates (significant “Location” effect; Table 1). In 2013 (post-spray sample), there was a 3-way interaction between “Treatment,” “Location,” and “Farm” (Table 1), indicating that the proportion of scarred fruit was higher in odor-baited bushes but this effect was influenced by both the proximity to the forest and the farm.

### 3.2. Does Fruit Injury Differ Between Bushes Neighboring Trap Bushes and Those Neighboring Control Bushes (Hypothesis 2a)?

**Figure 3:**
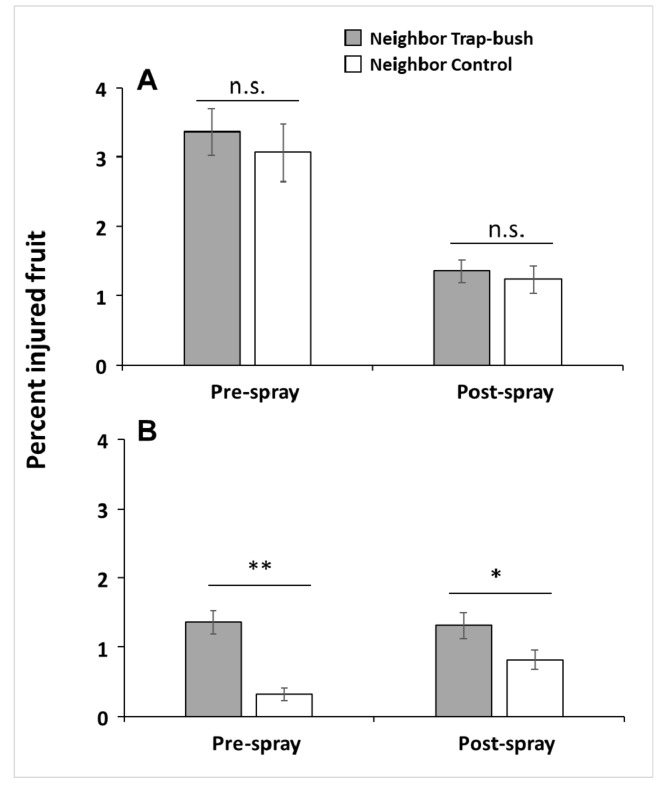
Percent (mean ± SE) of oviposition-scarred fruit by *Conotrachelus nenuphar* adults in unbaited bushes neighboring odor-baited trap bushes in trap-bush plots and in unbaited bushes neighboring unbaited bushes in control plots in 2013 (**A**) and 2014 (**B**). Fruit injury was assessed before and after a post-bloom insecticide application. Asterisks indicate significant differences according to Welch’s two-sample *t*-test (* = 0.01< *p* ≤ 0.05; ** = *p* ≤ 0.01); n.s. = not significant (*p* > 0.05).

Two analyses were conducted to address the question of a potential spillover effect, i.e., whether trap bushes increase levels of fruit injury in neighboring unbaited bushes. First, we compared the mean proportion of *C. nenuphar*-scarred fruit in bushes neighboring trap bushes with the mean proportion of scarred fruit in bushes neighboring control bushes. Here, if the proportion of scarred fruit is greater in baited neighbors than in unbaited neighbors then there is evidence for spillover. Although injured fruit was 10% higher in bushes neighboring trap bushes than those neighboring control bushes in 2013, this difference was not significant (pre-spray sample: Welch *t*-test_342.5_ = −0.57, *p* = 0.284; post-spray sample: Welch *t*-test_340.9_ = −0.49, *p* = 0.309) (Figure 3A). We found, however, significant evidence of spillover in 2014: injured fruit was 4.4 and 1.6 times higher in bushes neighboring trap bushes than those neighboring control bushes in both sampling dates, pre-spray (Welch *t*-test_291.5_ = −5.36, *p* < 0.001) and post-spray (Welch *t*-test_346.05_ = −2.06, *p* = 0.019), respectively (Figure 3B). These data indicate that unbaited bushes neighboring baited bushes tended to be more susceptible to injury by *C. nenuphar* than those near unbaited bushes, providing evidence for a potential spillover effect.

### 3.3. Does Fruit Injury Differ Between Trap Bushes and Their Neighboring Bushes (Hypothesis 2b)?

**Figure 4:**
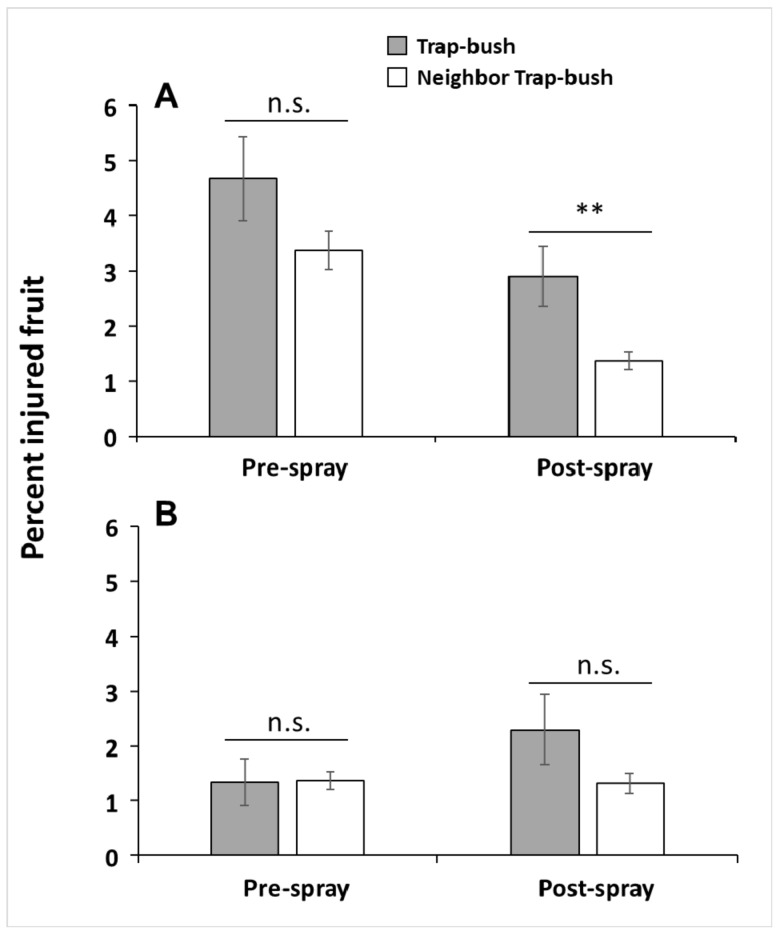
Percent (mean ± SE) of oviposition-scarred fruit by *Conotrachelus nenuphar* adults in trap bushes and in bushes neighboring trap bushes in 2013 (**A**) and 2014 (**B**). Fruit injury was assessed before and after a post-bloom insecticide application. Asterisks indicate significant differences according to Welch’s two-sample *t*-test (** = *p* ≤ 0.01); n.s. = not significant (*p* > 0.05).

We compared the mean proportion of *C. nenuphar*-scarred fruit in trap bushes with the mean proportion of scarred fruit in unbaited bushes neighboring trap bushes. In this analysis, no differences between these means indicate a possible spillover effect. In the 2013 post-spray sample, the mean proportion of scarred fruit was 2 times higher in trap bushes than in their neighboring bushes (Welch *t*-test_42.53_ = 2.72, *p* = 0.005) (Figure 4A). This difference was only marginally significant for the 2013 pre-spray sample (Welch *t*-test_51.69_ = 1.56, *p* = 0.063) and for the 2014 post-spray sample (Welch *t*-test_42.3_ = 1.47, *p* = 0.075) (Figure 4). There were, however, no differences in the proportion of scarred fruit between trap bushes and their neighboring bushes for the 2014 pre-spray sample (Welch *t*-test_48.66_ = −0.03, *p* = 0.512) (Figure 4B), or between unbaited bushes and their neighboring bushes for all sampling dates (all *p* values > 0.1). These results indicate that in 3 out of 4 sampling dates the proportion of scarred fruit was significantly or marginally higher in trap bushes than in their neighboring bushes, suggesting that injury by *C. nenuphar* in trap-bush plots tended to be greater in the baited bushes than in their neighbors.

### 3.4. Does Fruit Injury Differ Between Interior Bushes from Trap-tree Plots and Interior Bushes from Control Plots (Hypothesis 3)?

**Figure 5:**
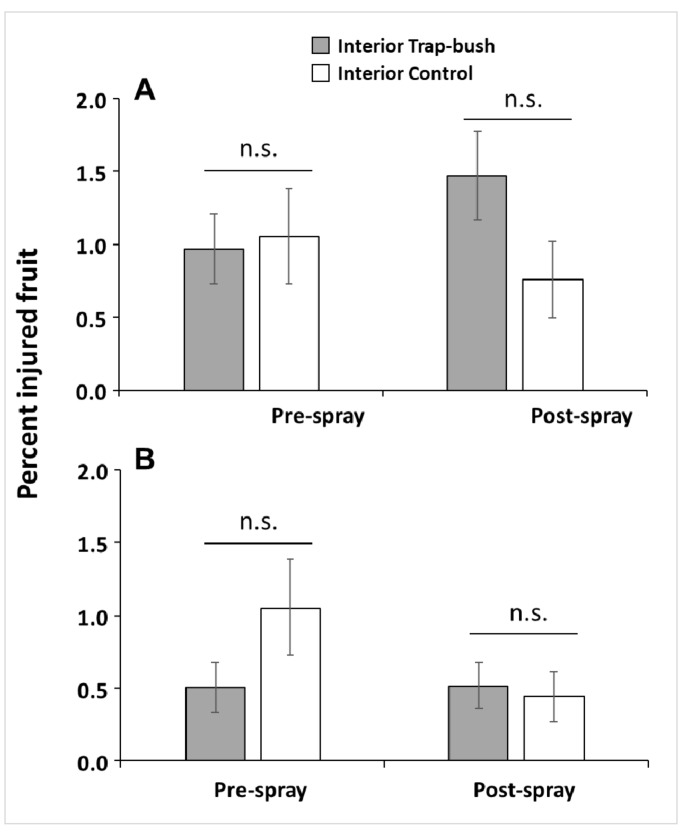
Percent (mean ± SE) of oviposition-scarred fruit by *Conotrachelus nenuphar* adults in interior trees within plots containing odor-baited trap bushes (trap-tree plots) or unbaited bushes (control plots) in 2013 (**A**) and 2014 (**B**). Fruit injury was assessed before and after a post-bloom insecticide application. n.s. = no significant differences according to Welch’s two-sample *t*-test (*p* > 0.05).

In general, fruit injury by *C. nenuphar* adults in all plot interiors was relatively low (<1.5%). In both years and sampling dates, there were no significant differences in the proportion of *C. nenuphar*-injured fruit between bushes from the interior of the trap-bush plots and the interior of control plots (2013 pre-spray sample: Welch *t*-test_130.1_ = 0.22, *p* = 0.415; 2013 post-spray sample: Welch *t*-test_125.7_ = −1.78, *p* = 0.961; 2014 pre-spray sample: Welch *t*-test_123.9_ = 1.15, *p* = 0.126; 2014 post-spray sample: Welch *t*-test_128.2_ = −0.29, *p* = 0.618) (Figure 5).

## 4. Discussion

This 2-year study demonstrated that: (1) commercial lures for *C. nenuphar* adults containing the aggregation pheromone grandisoic acid together with lures containing the host-plant volatile benzaldehyde successfully aggregate fruit injury when deployed within the canopy of highbush blueberries; (2) injury to fruit was greater in the baited bushes (referred to as trap bushes) than in unbaited bushes, but could also extend at least 3 bushes away (~9 m) from the baited bushes. As such, fruit injury among bushes could be ranked as follows: trap bushes ≥ neighboring trap bushes > unbaited bushes, providing some evidence for a spillover effect; and, (3) there was no difference in % infestation in fruit sampled from the interior of trap bush plots and from the interior of control plots.

Our study supports the hypothesis that baiting blueberry bushes with an attractive lure, i.e., a combination of aggregation pheromone and host-plant odors, results in aggregated oviposition injury by *C. nenuphar* adults. This method of luring weevils to specific host-plants, so-called trap tree, was successfully tested previously in apple orchards [20,21,22]. The trap-bush method developed here assumes that growers are willing to sacrifice some fruit from (perimeter) bushes due to injury. Similar to an “attract-and-kill” trap crop scenario [31], these bushes could then be specifically targeted for a post-bloom insecticide application [24], instead of applying an insecticide to an entire field; therefore saving application costs and providing an environmentally-friendlier tactic for managing this pest. In fact, Leskey et al. [24] reported a ~70% and 93% reduction in the total percentage of trees treated with insecticides under this trap-tree approach compared with perimeter row sprays and standard whole-orchard sprays, respectively, without compromising *C. nenuphar* control. In our study, growers were provided with recommendations on when to spray based on injury data. We basically compared two programs, one that relied solely on chemical control and one that, in addition to chemical control, uses trap bushes for monitoring *C. nenuphar* injury. In our case, chemical control consisted of a pre-bloom spray of novaluron (an insect growth regulator) and a post-bloom spray of phosmet (an organophosphate) to target three stages of the weevil, i.e., egg, larval, and adult growth and survival [28]. These programs resulted in a 3-fold reduction of fruit injury (to about 1%) in 2014. This is comparable to the level of fruit injury observed in apple orchards managed using the trap tree strategy or perimeter row treatments [24]. Needless to say, participating growers were very happy with our results. In fact, there is a zero tolerance for larval infestation for berries that are exported to other regions [12]; thus, management practices that closely achieve this goal are desirable to growers. However, recent studies have reported negative effects of novaluron on bee brood development [32,33,34], so care needs to be taken when using this insecticide pre-bloom. Targeting insecticide sprays to just the trap bushes and a few neighboring bushes could mitigate these negative effects on bees. As this insect pupates in the soil, there is also the possibility of integrating biological control through the use of entomopathogenic nematodes to target *C. nenuphar* larvae in the soil [35,36]. Targeted applications of nematodes to perimeter trap bushes could greatly reduce costs and provide an eco-friendly alternative to the current reliance on broad-spectrum insecticides. Integrating behavioral and biological control strategies constitutes a novel multi-faceted approach for managing *C. nenuphar* in highbush blueberries that may reduce the use of broad-spectrum insecticides while also providing options to organic blueberry growers who currently lack reliable control tools against this pest.

Location of the trap bush, whether along perimeter rows facing wooded habitats or along perimeter rows inside the field, did not seem to strongly influence the level of fruit injury by *C. nenuphar* adults. We expected to see higher oviposition activity along rows facing wooded habitat; however, this was true only in the 2013 pre-spray sampling. A likely explanation is that overwintered adults move into blueberry fields early in the spring prior to bloom, i.e., in April [9], while oviposition activity happens at fruit set, i.e., in mid- to late-May through early June. Hernandez-Cumplido et al. [17] reported an edge effect on adult captures in pyramid traps early in the season but this effect disappeared by May. Thus, by the time that susceptible fruits are available, *C. nenuphar* adults have already penetrated into the field interiors. This same phenomenon was reported by Piñero and Prokopy [37] in apple orchards in MA; on average 57% of *C. nenuphar* had entered orchards and potentially colonized apple trees by petal fall based on a 6-year study. Mark-recapture studies, like those done by Leskey and Wright [38], could help understand the response of *C. nenuphar* adults to trap bushes and how these odor-baited bushes affect their movement within blueberry fields. These data will aid at defining the size of a perimeter zone to target insecticide applications.

Our study also found some evidence of a spillover effect: unbaited blueberry bushes neighboring odor-baited trap bushes tended to have more fruit injury by *C. nenuphar* adults than those neighboring unbaited control bushes; although the level of injury in trap bushes tended to be higher than in their surrounding unbaited bushes. Prokopy et al. [21] noted that injury to trap trees was five-fold greater than in neighboring unbaited trees located 6–8 m away in apple orchards. Bushes in blueberry fields are, however, planted much closer together than trees in apple orchards. In fact, the distance between an odor-baited bush and its near neighboring unbaited bushes in our fields was ≤3 m, which means that the farthest distance between the trap bush and the unbaited neighboring bushes was ≤9 m (Figure 1). Our results are closely in line with the study of Leskey et al. [24] who found that injury around trap trees was greater than trees around unbaited (control) trees but found no difference between injury in the trap trees and injury in the nearest neighboring trees in apple orchards, indicative of spillover to trees adjacent to the trap trees. Altogether, our data suggest the potential of a spillover effect occurring at least 9 m from the trap bush in highbush blueberries. Recently, Hernandez-Cumplido et al. [17] found a greater number of *C. nenuphar* adults in blueberry bushes near odor-baited pyramid traps than unbaited traps early in the season but this aggregation of adults did not lead to greater oviposition injury to bushes later in the season. A likely explanation is that Hernandez-Cumplido et al. [17] used traps to collect and remove weevils from the blueberry fields prior to fruit set whereas in the present study weevils remained in the field which allowed them to move freely from trap bushes to neighboring unbaited bushes until a post-bloom insecticide application was allowed. Using mass trapping for *C. nenuphar* is impractical and cost prohibitive because of the size and price of current traps, as well as overall efficiency, particularly when developing fruit appears to compete with trap-based stimuli [38]. However, finding methods that limit weevil movement from trap bushes could help reduce spillover injury to adjacent unbaited bushes; thus, restricting insecticide use to even fewer bushes. It is possible, for example, to improve *C. nenuphar* attraction to, and retention on, trap bushes by improving the attractive lure (e.g., Reference [39]). Bee-safe methods to kill adults, such as insecticidal nets [40], could also be placed around the base of trap bushes for an “attract-and-kill” strategy to manage these weevils.

We predicted that trap bushes located around the perimeter of a blueberry field may reduce the level of fruit injury by *C. nenuphar* in the interior of fields, by retaining weevils in and around the trap bushes (i.e., plot perimeters) and thus reducing their numbers inside fields. We found that the amount of fruit injury did not differ between interior bushes in trap-bush plots and interior bushes in control plots. Similarly, Piñero et al. [22] and Leskey et al. [24] used perimeter-row, odor-baited trap trees to monitor oviposition activity and manage *C. nenuphar* adults in apple orchards and found no differences in levels of injury to fruit sampled from interior trees in plots managed under a trap tree regime, where insecticide applications were directed at trap trees only, and plots managed under conventional sprays or perimeter row sprays. Therefore, a similar (trap bush) strategy could be used in blueberries to monitor fruit injury or to manage *C. nenuphar* (i.e., attract-and-kill) without compromising fruit injury within fields.

## 5. Conclusions

This study examined the possibility of integrating behavioral and chemical control for the management of *C. nenuphar* in highbush blueberries. So far, programs for management of this pest have been limited to monitoring adults using ineffective, labor-intensive beat-sheet sampling methods and chemical control. Our study used trap bushes to monitor *C. nenuphar* injury to berries. Based on these data, participating growers responded to our 2013 recommendations with post-bloom insecticide applications that greatly decreased injury the subsequent year. Our results not only indicate that the trap bush method can be used to effectively monitor *C. nenuphar* injury but could also be used for site-specific applications to reduce insecticide use.

## Figures and Tables

**Table 1:** Statistical output for the main and interactive effects of baiting blueberry bushes with the aggregation pheromone grandisoic acid and the hostplant odor benzaldehyde (Treatment), their proximity to the forest (Location), and farm on the proportion of oviposition-scarred fruit by *Conotrachelus nenuphar* adults.

Sampling Year/Date ^1^	Variables ^2^	Df ^3^	*F*	*p* ^4^
2013/pre-spray	Treatment	1	0.58	0.451
	Location	1	12.72	**<0.001**
	Farm	3	2.18	0.101
	Treatment × Location	1	0.92	0.342
	Treatment × Farm	3	0.32	0.805
	Location × Farm	3	0.46	0.714
	Treatment × Location × Farm	3	1.11	0.352
2013/post-spray	Treatment	1	4.19	**0.046**
	Location	1	2.48	0.121
	Farm	3	4.20	**0.009**
	Treatment × Location	1	2.06	0.157
	Treatment × Farm	3	0.29	0.832
	Location × Farm	3	0.61	0.610
	Treatment × Location × Farm	3	4.28	**0.009**
2014/pre-spray	Treatment	1	8.45	**0.005**
	Location	1	0.00	0.985
	Farm	3	2.43	0.075
	Treatment × Location	1	0.06	0.802
	Treatment × Farm	3	1.54	0.216
	Location × Farm	3	0.53	0.665
	Treatment × Location × Farm	3	0.40	0.754
2014/post-spray	Treatment	1	5.18	**0.027**
	Location	1	0.33	0.569
	Farm	3	0.36	0.779
	Treatment × Location	1	0.48	0.492
	Treatment × Farm	3	0.85	0.475
	Location × Farm	3	0.79	0.506
	Treatment × Location × Farm	3	0.49	0.694

^1^ Data were analyzed separately for each year (2013 and 2014) and sampling date (pre- and post-insecticide spray). ^2^ Treatment and location were treated as fixed factors, while the effect of farm was treated as a random factor. ^3^ Residuals df = 51. ^4^ Significant *p* values (*p* ≤ 0.05) are shown in bold.
